# Hypoxemia in the ICU: prevalence, treatment, and outcome

**DOI:** 10.1186/s13613-018-0424-4

**Published:** 2018-08-13

**Authors:** SRLF Trial Group, D. Grimaldi, S. Hraiech, E. Boutin, J. C. Lacherade, F. Boissier, T. Pham, J. C. Richard, A. W. Thille, S. Ehrmann, J. B. Lascarrou, N. Aissaoui

**Affiliations:** Brussels, Belgium

**Keywords:** Hypoxemia, Epidemiology, Critical care, ARDS-acute respiratory failure, Invasive ventilation

## Abstract

**Background:**

Information is limited regarding the prevalence, management, and outcome of hypoxemia among intensive care unit (ICU) patients. We assessed the prevalence and severity of hypoxemia in ICU patients and analyzed the management and outcomes of hypoxemic patients.

**Methods:**

This is a multinational, multicenter, 1-day point prevalence study in 117 ICUs during the spring of 2016. All patients hospitalized in an ICU on the day of the study could be enrolled. Hypoxemia was defined as a PaO_2_/FiO_2_ ratio ≤ 300 mmHg and classified as mild (PaO_2_/FiO_2_ between 300 and 201), moderate (PaO_2_/FiO_2_ between 200 and 101), and severe (PaO_2_/FiO_2_ ≤ 100 mmHg).

**Results:**

Of 1604 patients included, 859 (54%, 95% CI 51–56%) were hypoxemic, 51% with mild (*n* = 440), 40% with moderate (*n* = 345), and 9% (*n* = 74) with severe hypoxemia. Among hypoxemic patients, 61% (*n* = 525) were treated with invasive ventilation, 10% (*n* = 84) with non-invasive ventilation, 5% (*n* = 45) with high-flow oxygen therapy, 22% (*n* = 191) with standard oxygen, and 1.6% (*n* = 14) did not receive oxygen. Protective ventilation was widely used in invasively ventilated patients. Twenty-one percent of hypoxemic patients (*n* = 178) met criteria for acute respiratory distress syndrome (ARDS) including 65 patients (37%) with mild, 82 (46%) with moderate, and 31 (17%) with severe ARDS. ICU mortality was 27% in hypoxemic patients and significantly differed according to severity: 21% in mild, 26% in moderate, and 50% in patients with severe hypoxemia, *p* < 0.001. Multivariate Cox regression identified moderate and severe hypoxemia as independent factors of ICU mortality compared to mild hypoxemia (adjusted hazard ratio 1.38 [1.00–1.90] and 2.65 [1.69–4.15], respectively).

**Conclusions:**

Hypoxemia affected more than half of ICU patients in this 1-day point prevalence study, but only 21% of patients had ARDS criteria. Severity of hypoxemia was an independent risk factor of mortality among hypoxemic patients.

*Trial registration* NCT 02722031

**Electronic supplementary material:**

The online version of this article (10.1186/s13613-018-0424-4) contains supplementary material, which is available to authorized users.

## Background

Hypoxemia is frequent and potentially life-threatening in critically ill patients. However, the prevalence, management, and outcomes of hypoxemia in non-selected intensive care unit (ICU) patients are poorly known. Most epidemiological studies have specifically focused on patients under mechanical ventilation [1–3] or those with criteria for acute respiratory distress syndrome (ARDS) [4–9], and none covered the entire spectrum of the whole hypoxemic patients. Whereas mechanical ventilation may be needed in non-hypoxemic patients, a number of hypoxemic patients are treated with oxygen while breathing spontaneously. The proportion of hypoxemic patients treated with standard or high-flow oxygen therapy and without need for mechanical ventilation is unknown among the whole patients in the ICU. Moreover, whereas the severity of hypoxemia was directly associated with mortality in patients with ARDS [10], this relation is not demonstrated among the other hypoxemic patients, especially in non-intubated patients. Finally, the relationship between respiratory support, hypoxemia severity, and outcome is not straightforward. It appears then important to provide epidemiological data regarding these gaps of knowledge that could help to design future trials on acute respiratory failure.

Therefore, we designed a large, multicenter, 1-day point prevalence study to measure the prevalence of hypoxemia (defined as a PaO_2_/FiO_2_ ratio ≤ 300 mmHg) among all ICU patients regardless of their oxygenation device and to stratify them according to the severity of hypoxemia (PaO_2_/FiO_2_ between 300 and 201 in mild, between 200 and 101 in moderate, and below or equal to 100 mmHg in severe). We also analyzed the potential impact of hypoxemia and its severity on outcome.

## Patients and methods

### Study design

The SPECTRUM study was a 1-day point prevalence study, endorsed by the French Intensive Care Society (Société de Réanimation de Langue Française: www.srlf.org) and conducted during the spring of 2016 in 117 ICUs in 7 French-speaking countries (62 general ICUs, 36 medical ICUs, 19 surgical ICUs). Additional file 1: Table S1 lists the participating centers and their characteristics. All patients already hospitalized in a participating ICU or newly admitted the day of the study could be enrolled. Exclusion criteria were refusal to participate and in France, being under guardianship, without social insurance, or being pregnant. Approval by the ethics committees according to the laws of each participating country was obtained (CE SRLF 15–35). In France, EC waived from written informed consent, and according to French law, the absence of opposition was obtained from patients or a next of kin before study enrollment.

### Definitions

Hypoxemia was defined as a PaO_2_/FiO_2_ ratio of 300 mmHg or less [5]. We stratified hypoxemia severity as mild for PaO_2_/FiO_2_ between 300 and 201 mmHg, moderate for PaO_2_/FiO_2_ between 200 and 101 mmHg, and severe for PaO_2_/FiO_2_ below or equal to 100 mmHg. ARDS was defined according to the Berlin definition [10]. Each single criterion of the ARDS definition was collected separately in the case report form. Patients fulfilling all the criteria were classified as having ARDS. Potential causes of ARDS were collected independently of the presence of hypoxemia and ARDS. Other causes/mechanisms of hypoxemia were also collected.

Oxygenation administration was classified as: no oxygen supply (ambient air), low-flow (conventional) oxygen whatever the device used (nasal cannula, mask, tracheostomy), high-flow oxygen through nasal cannula, non-invasive ventilation (NIV) whatever the modalities of ventilation, invasive ventilation through tracheal intubation or tracheostomy, and extracorporeal oxygenation. Patients with intermittent modes were classified with the mode in use at the time of data collection.

### Data collection

All data were assessed at the time of arterial blood gas measurement during the morning round. In cases where no arterial blood was drawn, data were recorded when the patient had an SpO_2_ ≤ 97%. Follow-up was restricted to ICU stay and censored at day 90 after the study day.

### PaO_2_/FiO_2_ determination

PaO_2_ and FiO_2_ were recorded simultaneously. In the case of low-flow oxygen without an FiO_2_ setting device, we calculated FiO_2_ as 0.21 + (oxygen flow rate in liters per minute × 0.03) [11]. In the absence of PaO_2_ measurement, we estimated the PaO_2_/FiO_2_ ratio through the SpO_2_/FiO_2_ ratio using the equation proposed by Rice et al. [12], using a SpO_2_ ≤ 97% because the Rice equation cannot infer PaO_2_ at higher SaO_2_ given the sigmoid relationship between SaO_2_ and PaO_2_.

In mechanically ventilated patients, we measured the following parameters: expired tidal volume (*V*_t_), measured respiratory rate, set positive end expiratory pressure (PEEP) and plateau pressure and PEEP during an inspiratory and expiratory pause in patients in volume control mode without spontaneous breathing. We calculated the driving pressure by subtracting total PEEP from plateau pressure [13].

### Statistical analysis

Prevalence of hypoxemia was computed using the total number of patients included as the denominator, and the 95% confidence interval was estimated (95% CI). We compared the three groups of hypoxemic patients with different degrees of severity in terms of baseline characteristics, comorbidities, and respiratory conditions. Quantitative variables were described as mean (SD) or median (25th–75th centiles), as appropriate, and compared using Student’s *t* test or the Wilcoxon–Mann–Whitney test. Qualitative variables were described as counts (%) and compared using the *χ*^2^ or the Fisher exact test, as appropriate. The completeness of the database is indicated in tables. No imputation was used for missing data.

Overall survival was estimated using the Kaplan–Meier method, and survival curves were compared according to the severity of hypoxemia using the log-rank test for categorical variables and the Wald test based on a univariate Cox model for quantitative variables. Among hypoxemic patients, Cox proportional hazards regression was performed to estimate unadjusted hazard ratios and their 95% CI. Variables associated with *p* values < 0.20 were selected for multivariable analyses. Confounders and interactions were tested in bivariate models. The proportional hazards assumption was assessed statistically using the Schoenfeld residuals test.

All tests were two-sided, and *p* values < 0.05 were considered significant. Analyses were conducted using Stata v12.1 (StataCorp LP, College Station, TX, USA).

The study was registered in Clinical trials: NCT 02722031.

## Results

### Characteristics of hypoxemic patients

**Fig. 1:**
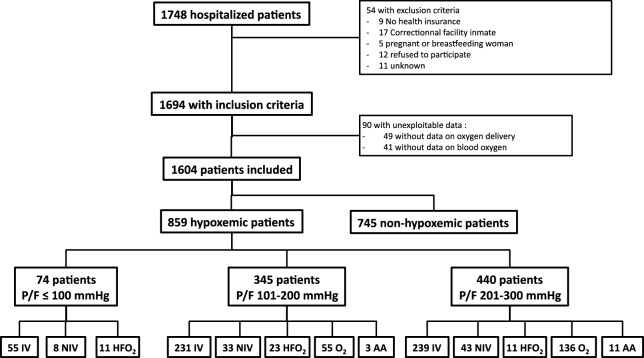
Flowchart of the study. *P*/*F*: PaO_2_/FiO_2_ ratio; IV: invasive ventilation, NIV non-invasive ventilation, HFO_2_: high-flow oxygen therapy, O_2_: low-flow (standard) oxygen therapy, AA: ambient air

Of the 1748 patients hospitalized in the participating ICUs the day of the study, 1604 were included. Flowchart of the study is given in Fig. 1. The general characteristics of the patients are reported in Table 1. The day of the study, 859 (54%, 95% CI 51–56%) patients were hypoxemic. Comparison between hypoxemic and non-hypoxemic patients is reported in Additional file 1: Table S2. Among the hypoxemic patients, 440 (51%) had mild, 345 (40%) moderate, and 74 (9%) severe hypoxemia. The causes and mechanisms of hypoxemia are given in Table 2. Pneumonia was the main cause of hypoxemia (53%), but there were a median of 2 [1–3] causes/mechanisms of hypoxemia per patient. A total of 178 (21%) patients fulfilled the criteria for ARDS: 65 (37%) had mild, 82 (46%) moderate, and 31 (17%) severe ARDS. ARDS exclusion criteria among the other 228 hypoxemic patients with bilateral infiltrates are shown in Additional file 1: Table S3. Even though the frequency of ARDS increased with severity of hypoxemia (*p* < 0.001), it represented less than 50% of severely hypoxemic patients (Table 2).

**Table 1:** Characteristics of hypoxemic patients according to severity of hypoxemia

	All hypoxemic patients, *n* = 859	Mild hypoxemia *P*/*F* 201–300 mmHg, *n* = 440	Moderate hypoxemia *P*/*F* 101–200 mmHg, *n* = 345	Severe hypoxemia *P*/*F* ≤ 100 mmHg, *n* = 74	*p*
Age, years median (IQR)	64 [53–73]	65 [54–74]	65 [53–73]	58 [47–69]	0.006
Female, *N* (%)	273 (31.8)	142 (32.3)	109 (31.6)	22 (29.7)	0.91
Body mass index (*n* = 844/431/341/72)	26.1 [22.5–31.1]	26.1 [22.7–30.9]	26.0 [22.5–31.0]	27.0 [22.1–34.4]	0.83
Main diagnosis at ICU admission (*n* = 858/439/345/74)					–
Septic shock	124 (14.5)	67 (15.2)	49 (14.2)	8 (10.8)	
Other shock	47 (5.5)	24 (5.4)	17 (4.9)	6 (8.1)	
Severe trauma	20 (2.3)	14 (3.2)	6 (1.7)	0 (0)	
De novo acute respiratory failure	275 (32.0)	118 (26.9)	120 (34.8)	37 (50.0)	
Acute on chronic respiratory failure	128 (14.9)	55 (12.5)	68 (19.7)	5 (6.8)	
Coma/seizures	66 (7.7)	43 (9.8)	19 (5.5)	4 (5.4)	
Metabolic disorders	18 (2.1)	11 (2.5)	6 (1.7)	1 (1.4)	
Hepatic failure	6 (0.7)	2 (0.5)	2 (0.6)	2 (2.7)	
Cardiac arrest	30 (3.5)	15 (3.4)	12 (3.5)	3 (4.1)	
Postoperative surveillance	96 (11.2)	64 (14.6)	31 (9.0)	1 (1.3)	
Other	48 (5.6)	26 (5.9)	15 (4.4)	7 (9.5)	
Admission category (*n* = 857/439/344/74)					0.004
Medical	675 (78.8)	327 (74.5)	281 (81.7)	67 (90.5)	
Scheduled surgery	76 (8.9)	49 (11.1)	27 (7.8)	0 (0)	
Urgent surgery	92 (10.7)	53 (12.1)	32 (9.3)	7 (9.5)	
Trauma	14 (1.6)	10 (2.3)	4 (1.2)	0 (0)	
SAPS II (*n* = 832/425/334/73)	43 [31–57]	42 [30–56]	43 [31–57]	45 [34–61]	0.32
Chronic respiratory disease (obstructive) (*n* = 855/440/342/73)	288 (33.7)	141 (32.1)	127 (37.1)	20 (27.4)	0.16
Chronic respiratory disease (restrictive) (*n* = 855/440/342/73)	71 (8.3)	28 (6.4)	37 (10.8)	6 (8.2)	0.08
Obstructive sleep apnea syndrome (*n* = 855/440/342/73)	71 (8.3)	36 (8.2)	29 (8.5)	6 (8.2)	0.99
Chronic oxygen therapy (*n* = 855/440/342/73)	71 (8.3)	32 (7.3)	33 (9.7)	6 (8.2)	0.49
Long-term non-invasive ventilation (*n* = 855/440/342/73)	32 (3.7)	17 (3.9)	14 (4.1)	1 (1.4)	0.68
Chronic heart failure (*n* = 855/440/342/73)	145 (17.0)	74 (16.8)	62 (18.1)	9 (12.3)	0.48
Chronic kidney failure (*n* = 855/440/342/73)	83 (9.7)	55 (12.5)	23 (6.7)	5 (6.9)	0.02
Cirrhosis (*n* = 855/440/342/73)	45 (5.3)	25 (5.7)	15 (4.4)	5 (6.9)	0.54
Cancer (*n* = 855/440/342/73)	79 (9.2)	43 (9.8)	32 (9.4)	4 (5.5)	0.50
Immunosuppression (*n* = 853/439/341/73)	99 (11.6)	46 (10.5)	43 (12.6)	10 (13.7)	0.55
Withholding/withdrawal of treatment the day of the study	105 (12.2)	41 (9.3)	47 (13.6)	17 (23)	0.002

**Table 2:** Mechanisms/causes of hypoxemia^a^ and radiological findings according to severity of hypoxemia on the day of the study

	All hypoxemic patients, *n* = 859	Mild hypoxemia, *n* = 440	Moderate hypoxemia, *n* = 345	Severe hypoxemia, *n* = 74	*p*
Pneumonia, *N* (%) (*n* = 852/440/339/73)	453 (53.2)	189 (43.0)	204 (60.2)	60 (82.2)	< 0.001
Aspiration (*n* = 852/440/339/73)	74 (8.7)	30 (6.8)	33 (9.7)	11 (15.1)	0.05
Acute on chronic respiratory failure (*n* = 853/440/340/73)	166 (19.5)	73 (16.6)	80 (23.5)	13 (17.8)	0.05
Cardiogenic pulmonary edema (*n* = 853/440/340/73)	127 (14.9)	64 (14.6)	51 (15.0)	12 (16.4)	0.91
Pulmonary embolism (*n* = 853/440/340/73)	26 (3.1)	16 (3.6)	7 (2.1)	3 (4.1)	0.37
Thoracic trauma (*n* = 852/440/339/73)	21 (2.5)	9 (2.1)	11 (3.2)	1 (1.4)	0.59
Pleural effusion (*n* = 852/440/339/73)	192 (22.5)	99 (22.5)	77 (22.7)	16 (21.9)	0.99
Atelectasis (*n* = 853/440/340/73)	176 (20.6)	93 (21.1)	72 (21.2)	11 (15.1)	0.47
Pneumothorax (*n* = 853/440/340/73)	15 (1.8)	6 (1.4)	7 (2.1)	2 (2.7)	0.49
Fluid overload (*n* = 853/440/340/73)	280 (32.8)	140 (31.8)	115 (33.8)	25 (34.3)	0.81
Acute pancreatitis (*n* = 852/440/339/73)	22 (2.6)	14 (3.2)	6 (1.8)	2 (2.7)	0.48
Shock, low PvO_2_ (*n* = 853/440/340/73)	110 (12.9)	42 (9.6)	48 (14.1)	20 (27.4)	< 0.001
Smoke/toxic inhalation (*n* = 852/440/339/73)	5 (0.6)	1 (0.2)	3 (0.9)	1 (1.4)	0.20
Transfusion-related acute lung injury (*n* = 852/440/339/73)	44 (5.2)	32 (7.3)	9 (2.7)	3 (4.1)	0.01
Pulmonary vasculitis (*n* = 852/440/339/73)	6 (0.7)	3 (0.7)	2 (0.6)	1 (1.4)	0.66
Drowning (*n* = 851/440/339/72)	1 (0.1)	1 (0.2)	0 (0)	0 (0)	1
Other (*n* = 851/440/338/73)	90 (10.6)	43 (9.8)	42 (12.4)	5 (6.9)	0.30
Number of causes of hypoxemia (*n* = 853^b^/440/340/73)	2 [1–3]	2 [1–3]	2 [1–3]	2 [1–3]	< 0.001
Number of causes of hypoxemia (*n* = 853^b^/440/340/73)					< 0.001
None	107 (12.5)	82 (18.6)	22 (6.5)	3 (4.1)	
1	230 (27.0)	110 (25.0)	100 (29.4)	20 (27.4)	
2	221 (25.9)	110 (25.0)	93 (27.3)	18 (24.7)	
3 or more	295 (34.6)	138 (31.4)	125 (36.8)	32 (43.8)	
Radiological infiltrates (*n* = 845/435/337/73)	583 (69.0)	257 (59.1)	260 (77.2)	66 (90.4)	< 0.001
Unilateral^c^ (*n* = 581/255/260/66)	176 (30.3)	85 (33.3)	81 (31.2)	10 (15.2)	0.02
Bilateral^c^ (*n* = 581/255/260/66)	406 (69.6)	171 (67.1)	179 (68.9)	56 (84.9)	0.02
ARDS	178 (20.7)	65 (14.7)	82 (23.8)	31 (41.9)	< 0.001

^a^Multiple causes and mechanisms could be selected

^b^In 6 patients data on hypoxemia, causes were not recorded

^c^Among patients with radiological infiltrates

Of the 1604 patients, 1110 (69%) had an arterial blood gas analysis (ABG) the day of the study. This proportion reached 87% (*n* = 749) in hypoxemic patients. ABG revealed that the most severely ill patients had the highest level of PaCO_2_ and the lowest level of PaO_2_ (Table 3).

**Table 3:** Respiratory conditions and outcome according to severity of hypoxemia

	All hypoxemic patients, *n* = 859	Mild hypoxemia *P*/*F* 201–300 mmHg, *n* = 440	Moderate hypoxemia *P*/*F* 101–200 mmHg, *n* = 345	Severe hypoxemia *P*/*F* ≤ 100 mmHg, *n* = 74	*p*
Respiratory rate (*n* = 846/432/340/74)	23 [18–27]	22 [18–26]	24 [20–28]	25 [20–30]	0.001
Arterial blood gases	749 (87.2)	368 (83.6)	311 (90.1)	70 (94.6)	0.004
pH (*n* = 745/366/309/70)	7.41 [7.36–7.46]	7.42 [7.38–7.45]	7.41 [7.36–7.46]	7.38 [7.29–7.45]	0.007
PaCO_2_ (mmHg) (*n* = 749/368/311/70)	41 [36–49]	40 [35–46]	43 [38–55]	45 [39–56]	0.001
PaCO_2_ > 45 mmHg (*n* = 749/368/311/70)	260 (34.7)	96 (26.1)	131 (42.1)	33 (47.1)	< 0.001
PaO_2_ (mmHg) (*n* = 749/368/311/70)	78 [66–92]	84 [73–100]	74 [64–88]	65 [56–74]	< 0.001
FiO_2_ (%) (*n* = 749/368/311/70)	0.40 [0.33–0.50]	0.35 [0.30–0.40]	0.50 [0.40–0.60]	1 [0.70–1]	< 0.001
HCO_3_-(meq/L) (*n* = 747/368/309/70)	26 [23–30]	26 [22–29]	27 [23–32]	26 [23–30]	0.03
Lactates (meq/L) (*n* = 630/312/257/61)	1.3 [1.0–1.9]	1.2 [0.9–1.7]	1.3 [1.0–1.9]	1.4 [1.1–2.7]	0.02
Oxygenation modalities, *N* (%)					< 0.001
Ambient air	14 (1.6)	11 (2.5)	3 (0.9)	0 (0)	
Low-flow oxygen	191 (22.2)	136 (30.9)	55 (15.9)	0 (0)	
High-flow oxygen	45 (5.2)	11 (2.5)	23 (6.7)	11 (14.9)	
Non-invasive ventilation	84 (9.8)	43 (9.8)	33 (9.6)	8 (10.8)	
Invasive ventilation	525 (61.2)	239 (54.3)	231 (66.9)	55 (74.3)	
Ventilator settings^a^					
Tidal volume, mL/kg of ideal body weight; median IQR (*n* = 509/230/225/54)	6.9 [6.1–7.9]	7.1 [6.2–8.1]	6.8 [6.0–7.6]	6.1 [4.8–6.6]	< 0.001
TV ≤ 8 mL/kg of ideal body weight (*n* = 509/230/225/54)	393 (77.2)	165 (71.7)	178 (79.1)	50 (92.6)	0.003
PEEP (cmH20) (*n* = 519/236/229/54)	6 [5–10]	5 [5–8]	7 [5–10]	10 [8–12]	< 0.001
Plateau pressure^b^ (cmH20) (*n* = 186/66/84/36)	22.5 [19–27]	20 [16–24]	23.5 [20–28]	25.5 [23–29.5]	< 0.001
Driving pressure^b^ (cmH20) (*n* = 186/66/84/36)	14 [10–18]	13 [9–17]	14 [11–18]	13 [10–19]	0.31
Adjunctive therapies					
Prone positioning (*n* = 516/232/230/54)	22 (4.3)	3 (1.3)	13 (5.7)	6 (11.1)	0.001
Inhaled NO (*n* = 513/233/226/54)	19 (3.7)	3 (1.3)	9 (4.0)	7 (13.0)	0.001
Continuous intravenous sedation, *N* (%) (*n* = 522/237/231/54)	321 (61.5)	121 (51.1)	159 (68.8)	41 (75.9)	< 0.001
Neuromuscular blocking agents, *N* (%) (*n* = 522/237/231/54)	83 (15.9)	11 (4.6)	44 (19.1)	28 (51.9)	< 0.001
Extracorporeal oxygenation, *N* (%) (*n* = 522/237/231/54)	26 (3.0)	5 (1.1)	7 (2.0)	14 (18.9)	< 0.001
ICU length of stay (days) (*n* = 840/431/336/73)	16 [7–32]	15 [7–32]	16 [8–31.5]	18 [7–37]	0.89
ICU length of stay (days) in ICU survivors (*n* = 625/342/246/37)	15 [7–30]	15 [7–30]	14 [6.5–30]	20.5 [9–46.5]	0.27
ICU mortality, *N* (%) (*n* = 839/431/335/73)	225 (26.7)	92 (21.3)	96 (28.5)	37 (50.7)	< 0.001

^a^Invasively ventilated patients *n* = 525

^b^Only in patients in whom plateau pressure was measurable (i.e., patients in VAC without spontaneous breathing). *n* = 186

Modalities of oxygen administration are reported in Table 3. Nearly 40% of the hypoxemic patients and 25% of the most severely ill were managed with non-invasive devices. Twenty-five per cent of patients on high-flow oxygen were severely hypoxemic, compared with less than 10% on NIV. Sixteen percent of these patients required later invasive ventilation in their ICU stay whatever the mode of support received the study day (Additional file 1: Figure S1). Causes and radiological finding according to the modalities of oxygen supply are depicted in Table 4. Adjunctive therapies designed to improve oxygenation beyond ventilation were mostly used in the severely hypoxemic patients, the most frequent being neuromuscular blockers, followed by extracorporeal membrane oxygenation, inhaled nitric oxide, and prone positioning (Table 3).

**Table 4:** Mechanisms/causes of hypoxemia and radiological findings according to oxygenation modalities

	Ambient air, *n* = 14	Low-flow oxygen, *n* = 191	High-flow oxygen, *n* = 45	Non-invasive ventilation, *n* = 84	Invasive ventilation, *n* = 525	*p*
Pneumonia, *N* (%) (*n* = 852/14/189/45/83/521)	3 (21.4)	62 (32.8)	34 (75.6)	37 (44.6)	317 (60.8)	< 0.001
Aspiration (*n* = 852/14/190/45/83/520)	0 (0)	11 (5.8)	0 (0)	3 (3.6)	60 (11.5)	0.003
Acute on chronic respiratory failure (*n* = 853/14/190/45/83/521)	0 (0)	32 (16.8)	4 (8.9)	41 (49.4)	89 (17.1)	< 0.001
Cardiogenic pulmonary edema (*n* = 853/14/190/45/83/521)	2 (14.3)	28 (14.7)	4 (8.9)	15 (18.1)	78 (15.0)	0.75
Pulmonary embolism (*n* = 853/14/190/45/83/521)	0 (0)	5 (2.6)	2 (4.4)	2 (2.4)	17 (3.3)	0.93
Thoracic trauma (*n* = 852/14/190/45/83/520)	0 (0)	5 (2.6)	2 (4.4)	0 (0)	14 (2.7)	0.46
Pleural effusion (*n* = 852/14/189/45/83/521)	2 (14.3)	34 (18.0)	11 (24.4)	24 (28.9)	121 (23.2)	0.3
Atelectasis (*n* = 853/14/190/45/83/521)	2 (14.3)	30 (15.8)	10 (22.2)	22 (26.5)	112 (21.5)	0.27
Pneumothorax (*n* = 853/14/190/45/83/521)	0 (0)	4 (2.1)	0 (0)	0 (0)	11 (2.1)	0.72
Fluid overload (*n* = 853/14/190/45/83/521)	2 (14.3)	47 (24.7)	12 (26.7)	28 (33.7)	191 (36.7)	0.02
Acute pancreatitis (*n* = 852/14/190/45/83/520)	0 (0)	6 (3.2)	0 (0)	1 (1.2)	15 (2.9)	0.81
Shock, low PvO_2_ (*n* = 853/14/190/45/83/521)	0 (0)	6 (3.2)	3 (6.7)	6 (7.2)	95 (18.2)	< 0.001
Smoke/toxic inhalation (*n* = 852/14/190/45/83/520)	0 (0)	0 (0)	0 (0)	0 (0)	5 (1.0)	0.63
Transfusion-related acute lung injury (*n* = 852/14/190/45/83/520)	1 (7.1)	10 (5.3)	2 (4.4)	3 (3.6)	28 (5.4)	0.92
Pulmonary vasculitis (*n* = 852/14/190/45/83/520)	0 (0)	1 (0.5)	0 (0)	0 (0)	5 (1.0)	1
Drowning (*n* = 851/14/190/45/82/520)	0 (0)	0 (0)	0 (0)	0 (0)	1 (0.2)	1
Other (*n* = 851/14/190/45/83/519)	0 (0)	21 (11.1)	11 (24.4)	9 (10.8)	49 (9.4)	0.04
Number of causes of hypoxemia (*n* = 853/14/190/45/83/521)	1 [0–1]	1 [1, 2]	2 [1, 2]	2 [1–3]	2 [1–3]	< 0.001
Number of causes of hypoxemia (*n* = 853/14/190/45/83/521)						< 0.001
None	6 (42.9)	41 (21.6)	0 (0)	6 (7.2)	54 (10.4)	
1	5 (35.7)	63 (33.1)	16 (35.6)	24 (28.9)	122 (23.4)	
2	2 (14.3)	45 (23.7)	18 (40.0)	23 (27.7)	133 (25.5)	
3 or more	1 (7.1)	41 (21.6)	11 (24.4)	30 (36.2)	212 (40.7)	
Radiological infiltrates (*n* = 845/14/189/45/82/515)	4 (28.6)	101 (53.4)	37 (82.2)	57 (69.5)	384 (74.6)	< 0.001
Unilateral c (*n* = 583/4/101/37/56/385)	1 (25.0)	36 (35.6)	9 (24.3)	19 (33.9)	111 (28.8)	0.58
Bilateral c (*n* = 581/4/100/37/56/384)	3 (75.0)	65 (65.0)	28 (75.7)	37 (66.1)	273 (71.1)	0.65
ARDS	0 (0)	0 (0)	0 (0)	9 (10.7)	169 (32.2)	< 0.001
Hypoxemia class						< 0.001
Mild	11 (78.6)	136 (71.2)	11 (24.4)	43 (51.2)	239 (45.5)	
Moderate	3 (21.4)	55 (28.8)	23 (51.2)	33 (39.3)	231 (44.0)	
Severe	0 (0)	0 (0)	11 (24.4)	8 (9.5)	55 (10.5)	
ICU length of stay (days) (*n* = 840/14/190/45/83/508)	11 [4–21]	8 [4–17]	14 [8–23]	9 [4–18]	21 [12–40]	< 0.001
ICU length of stay (days) in ICU survivors (*n* = 625/11/170/37/70/337)	11 [4–16]	7.5 [4–15]	13 [8–22]	9 [4–18]	24 [12–43]	< 0.001
ICU mortality, *N* (%) (*n* = 839/14/190/44/83/508)	3 (21.4)	20 (10.5)	7 (15.9)	13 (15.7)	168 (33.1)	< 0.001

### Ventilator settings in patients on invasive mechanical ventilation

Among hypoxemic patients under invasive mechanical ventilation, the measured median *V*_t_ was 6.9 [6.1–7.9] mL/kg of ideal body weight and 77% received a tidal volume of 8 mL/kg or less of ideal body weight. Tidal volume decreased with the severity of hypoxemia (Table 3). PEEP was set at 6 [5–10] cmH_2_O in the whole cohort and increased with the severity of hypoxemia to a median of 10 [8–12] cmH_2_O in the severely hypoxemic patients. Whereas plateau pressure increased with severity of hypoxemia, median driving pressure was below 15 cmH_2_O and similar in the 3 groups of patients (Table 3).

### Outcomes

Median ICU length of stay was 12 [5–28] days and ICU mortality was 20% in the 1604 patients of the study. In the hypoxemic population, median ICU length of stay was higher (16 [7–32] vs 8 [3–22] days in non-hypoxemic patients, *p* < 0.001) but not related to the severity of hypoxemia even when looking only at the patients leaving the ICU alive (Table 3). Patients with invasive ventilation had the longest stay in the ICU and the highest mortality (Table 4).

**Fig. 2:**
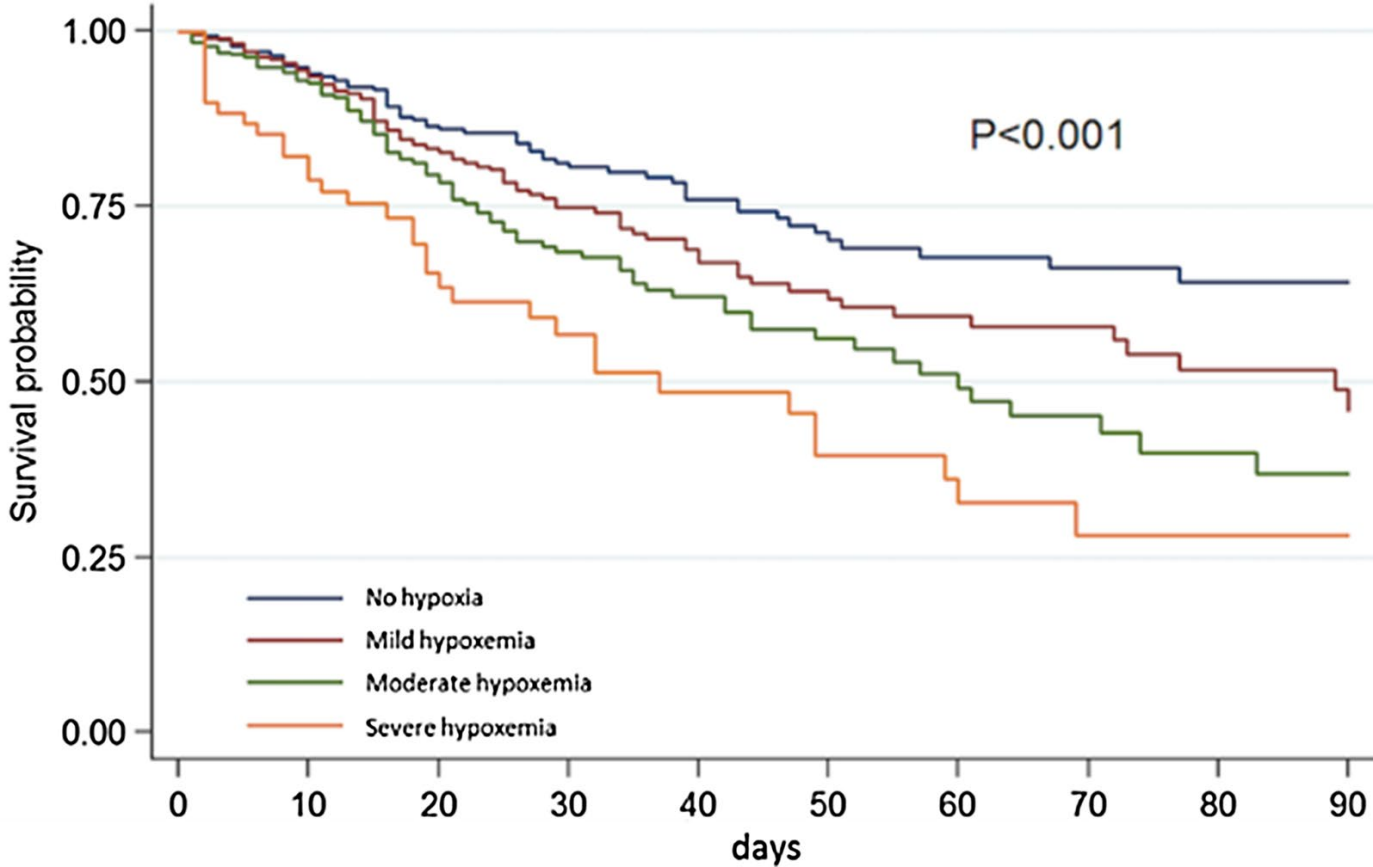
Survival curve according to hypoxemia severity. Survival curves were drawn according to the severity of hypoxemia using the Kaplan–Meier method and were compared using the log-rank test. Follow-up ended at the ICU leaving or was censored at day 90

ICU mortality was 12% in non-hypoxemic patients and 27% in hypoxemic patients (*p* < 0.001). ICU mortality increased with severity of hypoxemia: 21% in mildly, 29% in moderately, and 51% in severely hypoxemic patients (*p* < 0.001) (Table 3 and Fig. 2) and with invasiveness of ventilator support (Table 4). Additional file 1: Table S4 reports the ICU mortality according to ventilator support and hypoxemia class.

### Multivariate analysis

Taking into account the variables associated with ICU mortality in univariate analysis (Additional file 1: Table S5), multivariate analysis using a Cox model confirmed that moderate hypoxemia and severe hypoxemia were independently associated with ICU mortality compared to mild hypoxemia (adjusted hazard ratio 1.38 [1.00–1.90] and 2.65 [1.69–4.15], respectively), as well as other classic variables (Table 5). We performed several sensitivity analyses to strengthen this result (Additional file 1: Table S6): first, including the oxygen support in the analysis, second analyzing only patients with a PaO_2_/FiO_2_ ratio based on actual PaO_2_ and FiO_2_, third excluding patients on chronic oxygen therapy or chronic NIV, and fourth excluding patients with therapeutics withholding/withdrawing. These analyses show similar results, although moderate hypoxemia was no longer significantly associated with mortality (Additional file 1: Table S6). Moreover, analyzing the PaO_2_/FiO_2_ ratio as a continuous variable in the same model confirmed the independent association between the severity of hypoxemia and mortality (adjusted hazard ratio for each 10 mmHg decrease 1.06 [1.03–1.09], *p* < 0.001).

**Table 5:** Multivariate analysis of parameters associated with ICU survival

	Adjusted HR × [95% CI]	*p*
Age	1.01 [1.00–1.02]	0.054
Obesity	0.63 [0.45–0.89]	0.008
Main diagnosis at ICU admission		0.049
Septic shock	1.05 [0.68–1.64]	
Other shock	1.83 [1.09–3.07]	
Severe trauma	0.29 [0.04–2.13]	
De novo acute respiratory failure	1 (ref)	
Acute on chronic respiratory failure	1.56 [0.95–2.57]	
Coma/seizures	1.58 [0.89–2.78]	
Metabolic disorders	0.74 [0.25–2.19]	
Cardiac arrest	1.73 [0.88–3.40]	
Postoperative surveillance	0.67 [0.32–1.40]	
Other	0.76 [0.37–1.56]	
Chronic heart failure	1.69 [1.19–2.42]	0.004
Chronic kidney failure	1.65 [1.05–2.62]	0.032
Cirrhosis	1.71 [0.95–3.10]	0.075
SAPS II (per point)	1.01 [1.00–1.02]	0.012
Hypoxemia class		< 0.001
Mild	1 (ref)	
Moderate	1.38 [1.00–1.90]	
Severe	2.65 [1.69–4.15]	

*SAPS II* Simplified Acute Physiology Score

Multivariate Cox analysis of variables associated with ICU survival

## Discussion

This large, multicenter study gives for the first time data on the prevalence of hypoxemia in non-selected ICU-hospitalized patients. Strikingly, though more than half of the patients hospitalized in ICUs the day of the study were hypoxemic, 79% did not fulfill the criteria of ARDS according to the Berlin definition [10]. The prevalence of ARDS, albeit greater in the most hypoxemic patients, was still less than half (42%). Our results highlight also that hypoxemia is frequent among non-ventilated patients. The other main result of our study is that among hypoxemic patients, moderate and severe hypoxemia compared to mild hypoxemia was independently associated with higher ICU mortality.

Data concerning hypoxemia in unselected ICU patients, regardless of the presence of ARDS or the use of mechanical ventilation, are rather scarce, and we think this is strength of the SPECTRUM study to have included patients with all types of oxygenation devices. In the LUNG-SAFE study, hypoxemia was less frequent (35%), but all the patients considered in the analysis received ventilatory support [4], unlike those analyzed in our study. In another study focusing on ARDS [5], the incidence of hypoxemia was 50% among ventilated patients, but no data on non-invasively ventilated patients were given.

The prevalence of ARDS in the SPECTRUM study was 21% in hypoxemic patients, leading to an overall prevalence of 11% of included patients. This prevalence rate is close to the incidence found in the LUNG-SAFE study, in which 10.4% of the patients admitted in the ICU (23% of the mechanically ventilated patients) met the criteria for ARDS. Other studies, using the 1994 ALI/ARDS definition, reported similar results [5] or higher incidence [7, 8].

The presence of hypoxemia was associated with higher mortality and ICU length of stay, and the association with mortality was even stronger in the group of patients with severe hypoxemia. Severe hypoxemia was still associated with mortality after adjustment on respiratory support and in several sensitivity analyses, strengthening this result. This association is not so straightforward as several interventions known to improve PaO_2_/FiO_2_ ratio failed to reduce mortality [14] or were even detrimental [15, 16], suggesting that improving PaO_2_/FiO_2_ ratio cannot be an isolated therapeutic goal. Interestingly, ICU mortality among patients with mild-to-moderate hypoxemia was lower than that reported in patients with mild-to-moderate ARDS [4]. Conversely, mortality in our severely hypoxemic patients was close to that of severe ARDS patients described in other large studies. This result may be due to the fact that ARDS accounted for almost half of the severely hypoxemic patients in our cohort but also to the fact that similar pathophysiological mechanisms may be involved in severe hypoxemic patients with or without ARDS. For instance, pneumonia and aspiration as a cause of hypoxemia were more frequent in the most severe patients compared to other frequent causes of hypoxemia (see Table 2). Further studies should try to decipher the respective roles of ARDS and hypoxemia in the prognosis.

Invasiveness of respiratory support was also associated with mortality in univariate analysis, but time-dependent multivariate analysis did not confirm this association. Indeed, roles of respiratory support and severity of hypoxemia in mortality have a complex interplay as the most severe patients received the more invasive support and as respiratory support is not fixed across disease course. Our data suggest that severity of hypoxemia assessed by the *P*/*F* ratio a given day is associated with mortality whatever the ventilator support needed in line with other study [17]. This finding may be useful in clinical practice to assess even roughly the severity of hypoxemia and the potential impact on outcomes.

We also evaluated the oxygenation devices used and the ventilator settings in hypoxemic patients. High-flow oxygen and NIV were not rare, even among severely hypoxemic patients. Almost 20% of the patients with a PaO_2_/FiO_2_ ratio ≤ 100 were not on invasive mechanical ventilation, despite the fact that several studies have shown that NIV for de novo hypoxemic respiratory failure is associated with a poorer outcome in patients who finally required invasive mechanical ventilation [18–20]. We included all patients in the ICU and presenting hypoxemia (both de novo acute respiratory failure and acute on chronic respiratory failure), and it may be that those patients with severe hypoxemia on NIV had severe chronic respiratory failure or severe cardiogenic pulmonary edema. High-flow oxygen therapy was recently shown to have beneficial effects in patients with a PaO_2_/FiO_2_ ratio ≤ 200, as compared with standard oxygen and NIV in a randomized controlled trial [11]. However, the outcome of patients with the most severe hypoxemia receiving high-flow oxygen is unknown. Interestingly, in our study, high-flow oxygen was proportionally more frequently used in the most severely ill patients.

In mechanically ventilated patients, hypoxemia was associated with the use of “protective ventilation” in most cases with low *V*_t_ and higher PEEP, permissive hypercapnia and hypoxemia resulting in median plateau and driving pressures below the commonly admitted thresholds. Whereas reduced tidal volume appeared beneficial in observational studies among non-ARDS patients [21] and is currently the object of ongoing trials, the value of other protective strategies such as reducing driving pressure, increasing PEEP, or prone positioning may deserve evaluation in this population. The use of adjunctive therapies was correlated with the degree of hypoxemia. Neuromuscular blocking agents were the most often used in line with recent ARDS guidelines [22]: half of the patients with a PaO_2_/FiO_2_ ratio ≤ 100 were continuously paralyzed. More surprising is the limited use of prone positioning on the day of the study in the subgroup of severely hypoxemic patients, even if not all patients fulfilled ARDS criteria. Recourse to prone positioning was, for instance, less frequent than the use of inhaled NO, whereas the level of evidence widely favors prone positioning rather than inhaled NO during ARDS [23–25].

Our study provides novel data about hypoxemia prevalence and management that could have implications for research and clinical practice. We provide an accurate prevalence of hypoxemia and stratification according to its severity even in those breathing spontaneously. This could help in the design of future interventional studies targeting these patients. In the clinical practice, physician should be alerted by the severity of hypoxemic non-intubated patients and the risk of mortality.

As we enrolled more than 90% of the screened patients, our observations were likely unbiased. However, our study has some limits. First, it was a point prevalence study and thus does not yield accurate data on incidence. By design, patients with prolonged stay had more chance to be included and are thus overrepresented in our study. Data on length of stay had then to be interpreted with caution and does not reflect accurately the mean length of stay of patients admitted in the ICU. Given the higher ICU length of stay of hypoxemic patients, one could hypothesize that the incidence of hypoxemia is lower than the prevalence. Conversely, some patients without hypoxemia on the day of study may have had hypoxemia before or after the study day during their ICU stay, and some hypoxemic patients may have been more severe. Likewise, our data on adjuvant therapies should be analyzed with caution as some, such as extracorporeal membrane oxygenation, cover a long period of time and so increase the chance of being present on the day of the study. Second, arterial blood gas was not analyzed in 31% of patients. In those cases, hypoxemia was assessed with an extrapolation of the PaO_2_/FiO_2_ ratio from the SpO_2_/FiO_2_ ratio. However, most patients classified as “hypoxemic” underwent arterial blood gas analysis on the day of the SPECTRUM study. Moreover, published data show that PaO_2_/FiO_2_ can be accurately derived from the SpO_2_/FiO_2_ ratio [12, 26, 27] and sensitivity analysis including only patients with arterial blood gases was consistent with our result on mortality. Third, we conducted our study in spring to avoid the inclusion during an influenza seasonal pandemic; thus, our results could have been different in other seasons. Fourth, the inclusion of patients with limitation of treatment could impact our results as invasive devices are less likely to be used and mortality is higher in these patients. However, this allows to report in an unbiased way our epidemiological data. Lastly, the difficulty of separating ARDS from non-ARDS in hypoxemic patients is well known. In our survey, the diagnosis of ARDS was based on the Berlin criteria given in the CRF, independently of the clinical judgment of the clinician. However, chest-X ray analysis was not independently assessed. Even though we found a percentage of ARDS similar to that found in other large cohorts, it has been shown that ARDS is largely underdiagnosed, especially in its mild forms [4]. Even in the era of the Berlin definition, ARDS continues to be underrecognized, especially because of concerns about the reliable interpretation of chest radiographs [28]. Finally, although we identified an independent association between hypoxemia and mortality, our study does not provide mechanistic explanations of this finding.

## Conclusion

In this 1-day point prevalence study, more than half of the patients suffered from hypoxemia, of mostly mild-to-moderate severity. ARDS criteria were present the day of data collection in only one out of five patients and were far from capturing the spectrum of hypoxemia in the ICU setting. Non-invasive methods of oxygenation were largely used even in the most hypoxemic patients. Finally, among hypoxemic patients, moderate and severe hypoxemia were independently associated with ICU survival, as compared to mild hypoxemia.

## Additional file

**Additional file 1:** **Table S1.** List of contributors; **Table S2.** Patients’ characteristics according to the presence of hypoxemia; **Table S3.** ARDS exclusion criteria among patients with bilateral infiltrates; **Table S4.** ICU mortality according to ventilator support and hypoxemia class; **Table S5.** Univariate analysis of variables associated with mortality in hypoxemic patients; **Table S6.** aHRs of PaO2 / FiO2 banding in different sensitivity analyses; **Figure S1.** Evolution of oxygen support after the day of study.
