# Correction to: Hypoxemia in the ICU: prevalence, treatment, and outcome

**DOI:** 10.1186/s13613-019-0486-y

**Published:** 2019-01-21

**Authors:** SRLF Trial Group, Nadia Aissaoui, Laurent Argaud, Cécile Aubron, Julio Badie, Gaetan Beduneau, Florence Bellenfant, Pascal Beuret, Jeremy Bourenne, Guillaume Carteaux, Bertrand Canoville, Serge Carreira, Sébastian Champion, Sébastien Jochmans, Franck Chemouni, Karim Chergui, Akli Chermak, Stéphanie Chevalier, Bernard Cholley, Anne Courte, Mircea Cristinar, Daniel Da Silva, Roux Damien, Marc Danguy Des Déserts, Diane Commandeur, Pierre Eric Danin, Pierre-Yves Delannoy, Jean Dellamonica, Alexandre Demoule, Danielle Reuter, Hélène Prodanovic, Nicolas Deye, Pierre Yves Egreteau, Stephan Ehrmann, Etienne Escudier, Martine Ferrandiere, Jérôme Fichet, Bruno Filloux, Arnaud Galbois, Josette Gally, Aude Garnero, Jean-François Georger, Marie Luce Gilbert, Antoine Gros, Mathieu Guilbart, Guillaume Franchineau, Max Guillot, Olivier Guisset, Clément Hoffmann, Sami Hraiech, Julien Huntzinger, Dominique Hurel, Frederic Jacobs, Bernard Just, Clément Dubost, Kevin Kearns, Antoine Kimmoun, Camille Alzina, Jean-Claude Lacherade, Karim Lakhal, Christian Lamer, Mickael Landais, Cécile Lory, Guillaume Louis, Fouad Fadel, Philippe Michel, Nathalie Marin, Abolfazl Mohebbi Amoli, Grégoire Muller, Alexandru Nica, Gaetan Plantefeve, Fabienne Plouvier, Jean Baptiste Putegnat, Jean-Pierre Quenot, Jean Reignier, Jack Richecoeur, Jean-Philippe Rigaud, Adrien Robine, Marc Leone, Clément Olivier, David Sauvajon, Guillaume Schnell, Alexis Soummer, Annabelle Stoclin, Nicolas Terzi, Arnaud W Thille, Florence Boissier, Martial Thyrault, Amaury Toitot, Alexandre Tonnelier, Jean Turc, François Vincent, Nicolas Weiss, Phillipe Durasnel, Guillaume Thiery, Arnaud Winer, David Couret, Richard Galliot, Mathieu Série, Stéphanie Houcke, Jean Catineau, Dimitri De Wilde, Philippe Dechamps, David Grimaldi, Michael Piagnerelli, Antonio Artigas, Ferran Roche-Campo, Frank Gils, Philippe Jouvet, Emidio Jorge Santos Lima, Kamel Aissi, Aminé Ali Zeggwagh, Hanane Ezzouine, Jalila Benkhelil, Salah Benlakhal, Lamia Besbes, Mohamed Boussarsar, Rim Gharbi, Ines Sedghiani, Zouheir Jerbi, Mathieu Mattei, Jérémie Lemarié, Thierry Soupison, Virginie Williams, Stéphane Delisle, Emmanuel Charbonney, Tài Pham, Jean-Christophe Richard, Florence Brouard, Saber Barbar, Gaël Piton, Jean-Baptiste Lascarrou, Emmanuelle Boutin

**Affiliations:** Brussels, Belgium

## Correction to: Intensive Care (2018) 8:82 10.1186/s13613-018-0424-4

Following publication of the original article [1], we have been notified that only the 11 members of the writing committee are listed as the collaborators of the SRLF Trial group.

In this correction article, all members of the SRLF Trial group are now correctly shown in the collaborator list and all members are listed in Additional file 1 for reference. The online and pdf versions of this paper are not affected by the change.

The published apologizes for any inconvenience caused by this error.

## Additional file

**Additional file 1: Table S1.** List of contributors; **Table S2.** Patients’ characteristics according to the presence of hypoxemia; **Table S3.** ARDS exclusion criteria among patients with bilateral infiltrates; **Table S4.** ICU mortality according to ventilator support and hypoxemia class; **Table S5.** Univariate analysis of variables associated with mortality in hypoxemic patients; **Table S6.** aHRs of PaO2/FiO2 banding in different sensitivity analyses; **Figure S1.** Evolution of oxygen support after the day of study.
