# Supplementary material for: Hypoxemia in the ICU: prevalence, treatment, and outcome
Source: Ann Intensive Care. 2018 Aug 13;8:82. doi: 10.1186/s13613-018-0424-4 (PMC6089859; doi:10.1186/s13613-018-0424-4)
Supplement: Supplementary file 1 — Additional file 1: Table S1. List of contributors; Table S2. Patients’ characteristics according to the presence of hypoxemia; Table S3. ARDS exclusion criteria among patients with bilateral infiltrates; Table S4. ICU mortality according to ventilator support and hypoxemia class; Table S5. Univariate analysis of variables associated with mortality in hypoxemic patients; Table S6. aHRs of PaO2 / FiO2 banding in different sensitivity analyses; Figure S1. Evolution of oxygen support after the day of study. [file 13613_2018_424_MOESM1_ESM.docx]

Electronic Supplementary Material: Spectrum study from the SRLF trial group

**Additional file 1: Table S1.  List of contributors**

| **Last name** | **First name** | **City** | **Country** | **Department** |
| --- | --- | --- | --- | --- |
| Aissaoui | Nadia | Paris | France | medical ICU |
| Argaud | Laurent | Lyon | France | medical ICU |
| Aubron | Cécile | Brest | France | medical ICU |
| Badie | Julio | Belfort | France | medical ICU |
| Beduneau | Gaetan | Rouen | France | medical ICU |
| Bellenfant | Florence | Paris | France | surgical ICU |
| Beuret | Pascal | Roanne | France | medical ICU |
| Bourenne | Jeremy | Marseille | France | general ICU |
| Carteaux | Guillaume | Créteil | France | medical ICU |
| Canoville | Bertrand | Caen | France | medical ICU |
| Carreira | Serge | Bry Sur Marne | France | general ICU |
| Champion | Sébastian | Le Chesnay | France | Cardiac ICU |
| Jochmans | Sébastien | Melun | France | general ICU |
| Chemouni | Franck | Jossigny | France | general ICU |
| Chergui | Karim | Corbeil-Essonnes | France | general ICU |
| Chermak | Akli | Etampes | France | Respiratory ICU |
| Chevalier | Stéphanie | Saint Malo | France | medical ICU |
| Cholley | Bernard | Paris | France | cardiac ICU |
| Courte | Anne | Saint Brieuc | France | medical ICU |
| Cristinar | Mircea | Strabourg | France | cardiac ICU |
| Da Silva | Daniel | Saint-Denis | France | general ICU |
| Damien | Roux | Colombes | France | general ICU |
| Danguy Des Déserts | Marc | Brest | France | Surgical ICU |
| Commandeur | Diane | Brest | France | Surgical ICU |
| Danin | Pierre Eric | Nice | France | digestive ICU |
| Delannoy | Pierre-Yves | Tourcoing | France | general ICU |
| Dellamonica | Jean | Nice | France | medical ICU |
| Demoule | Alexandre | Paris | France | medical ICU |
| Reuter | Danielle | Paris | France | medical ICU |
| Prodanovic | Hélène | Paris | France | medical ICU |
| Deye | Nicolas | Paris | France | medical ICU |
| Egreteau | Pierre Yves | Morlaix | France | general ICU |
| Ehrmann | Stephan | Tours | France | general ICU |
| Escudier | Etienne | Annecy | France | general ICU |
| Ferrandiere | Martine | Tours | France | Surgical ICU |
| Fichet | Jérôme | Saint-Denis | France | cardiac ICU |
| Filloux | Bruno | Cornebarrieu | France | general ICU |
| Galbois | Arnaud | Quincy-Sous-Sénart | France | medical ICU |
| Gally | Josette | Mulhouse | France | general ICU |
| Garnero | Aude | Toulon | France | general ICU |
| Georger | Jean-François | Villeneuve St-George | France | general ICU |
| Gilbert | Marie Luce | Cahors | France | general ICU |
| Gros | Antoine | Le Chesnay | France | general ICU |
| Guilbart | Mathieu | Amiens | France | cardiac ICU |
| Franchineau | Guillaume | Paris | France | medical ICU |
| Guillot | Max | Strasbourg | France | medical ICU |
| Guisset | Olivier | Bordeaux | France | medical ICU |
| Hoffmann | Clément | Clamart | France | surgical ICU |
| Hraiech | Sami | Marseille | France | medical ICU |
| Huntzinger | Julien | Vannes | France | general ICU |
| Hurel | Dominique | Mantes La Jolie | France | general ICU |
| Jacobs | Frederic | Clamart | France | medical ICU |
| Just | Bernard | Charleville-Mezieres | France | general ICU |
| Dubost | Clément | Saint-Mande | France | general ICU |
| Kearns | Kevin | Saint-Mande | France | general ICU |
| Kimmoun | Antoine | Nancy | France | medical ICU |
| Alzina | Camille | Mulhouse | France | General ICU |
| Lacherade | Jean-Claude | La Roche Sur Yon | France | general ICU |
| Lakhal | Karim | Nantes | France | surgical ICU |
| Lamer | Christian | Paris | France | surgical ICU |
| Landais | Mickael | Le Mans | France | general ICU |
| Lory | Cécile | Guéret | France | general ICU |
| Louis | Guillaume | Metz | France | general ICU |
| Fadel | Fouad | Pontoise | France | general ICU |
| Michel | Philippe | Beaumont Sur Oise | France | general ICU |
| Marin | Nathalie | Paris | France | Medical ICU |
| Mohebbi Amoli | Abolfazl | Anthony | France | general ICU |
| Muller | Grégoire | Orléans | France | general ICU |
| Nica | Alexandru | Compiegne | France | general ICU |
| Plantefeve | Gaetan | Argenteuil | France | general ICU |
| Plouvier | Fabienne | Agen | France | general ICU |
| Putegnat | Jean Baptiste | Lyon | France | general ICU |
| Quenot | Jean-Pierre | Dijon | France | medical ICU |
| Reignier | Jean | Nantes | France | medical ICU |
| Richecoeur | Jack | Beauvais | France | general ICU |
| Rigaud | Jean-Philippe | Dieppe | France | medical ICU |
| Robine | Adrien | Bourg En Bresse | France | general ICU |
| Leone | Marc | Marseille | France | Surgical ICU |
| Olivier | Clément | Angers | France | Medical ICU |
| Sauvajon | David | Chambéry | France | medical ICU |
| Schnell | Guillaume | Le Havre | France | general ICU |
| Soummer | Alexis | Suresnes | France | general ICU |
| Stoclin | Annabelle | Villejuif | France | general ICU |
| Terzi | Nicolas | Grenoble | France | medical ICU |
| Thille | Arnaud W | Poitiers | France | Medical ICU |
| Boissier | Florence | Poitiers | France | Medical ICU |
| Thyrault | Martial | Longjumeau | France | general ICU |
| Toitot | Amaury | Lons Le Saunier | France | general ICU |
| Tonnelier | Alexandre | Quimper | France | general ICU |
| Turc | Jean | Lyon | France | general ICU |
| Vincent | François | Montfermeil | France | medical ICU |
| Weiss | Nicolas | Paris | France | neuro-ICU |
| Durasnel | Phillipe | Mamoudzou | Mayotte | General ICU |
| Thiery | Guillaume | Pointe À Pitre | France | General ICU |
| Winer | Arnaud | Saint Pierre - La Réunion | France | General ICU |
| Couret | David | Saint Pierre - La Réunion | France | Neuro-ICU |
| Galliot | Richard | Saint Pierre - La Réunion | France | General ICU |
| Série | Mathieu | Nouvelle-Calédonie | France | General ICU |
| Houcke | Stéphanie | Cayenne-Guyane | France | Medical ICU |
| Catineau | Jean | Monaco | Monaco | General ICU |
| De Wilde | Dimitri | Herentals | Belgium | General ICU |
| Dechamps | Philippe | Bruxelles | Belgium | General ICU |
| Grimaldi | David | Bruxelles | Belgium | General ICU |
| Piagnerelli | Michael | Charleroi | Belgium | General ICU |
| Artigas | Antonio | Sabadell | Spain | General ICU |
| Roche-Campo | Ferran | Tortosa | Spain | General ICU |
| Gils | Frank | Luxembourg | Luxembourg | General ICU |
| Jouvet | Philippe | Montreal | Canada | General ICU |
| Jorge Santos Lima | Emidio | Salvador | Salvador | General ICU |
| Aissi | Kamel | Annaba | Algeria | General ICU |
| Zeggwagh | Aminé Ali | Rabat | Marocco | Neuro-ICU |
| Ezzouine | Hanane | Casablanca | Marocco | General ICU |
| Benkhelil | Jalila | Ariana | Tunisia | medical ICU |
| Benlakhal | Salah | Tunis | Tunisia | medical ICU |
| Besbes | Lamia | Monastir | Tunisia | general ICU |
| Boussarsar | Mohamed | Sousse | Tunisia | medical ICU |
| Gharbi | Rim | Mahdia | Tunisia | medical ICU |
| Sedghiani | Ines | Tunis | Tunisia | General ICu |
| Jerbi | Zouheir | Tunis | Tunisia | General ICU |
| Mattei | Mathieu | Nancy | France | Cardiac ICU |
| Lemarié | Jérémie | Nancy | France | General ICU |
| Soupison | Thierry | Amiens | France | Medical ICU |
| Williams | Virginie | Montreal | Canada | General ICU |
| Delisle | Stéphane | Montreal | Canada | General ICU |
| Charbonney | Emmanuel | Montréal | Canada | General ICU |
| Pham | Tài | Toronto | Canada | General ICU |
| Richard | Jean-Christophe | Lyon | France | Medical ICU |
| Brouard | Florence | Perigueux | France | General ICU |
| Barbar | Saber | Nimes | France | General ICU |
| Piton | Gaël | Besançon | France | Medical ICU |
| Lascarrou | Jean-Baptiste | Nantes | France | Medical ICU |
| Boutin | Emmanuelle | Creteil | France | Statistic |

**Additional file 1: Table S2.  Patients’ characteristics according to the presence of hypoxemia**

|  | Total n=1604 | Non-hypoxemic n=745 | Hypoxemic n=859 | p |
| --- | --- | --- | --- | --- |
|  |  |  |  |  |
| **Age (n=1602/743/859)** | 63 [51-73] | 62 [48-72] | 64 [53-73] | 0.001 |
| Female | 585 (36.5) | 312 (41.9) | 273 (31.8) | <0.001 |
| **BMI kg/m² (n=1571/727/844)** | 25.7 [22.3-30.6] | 25.3 [22.0-30.0] | 26.1 [22.5-31.1] | 0.02 |
| **Obesity (n=1571/727/844)** | 428 (27.2) | 181 (24.9) | 247 (29.3) | 0.05 |
| **Main diagnosis at ICU admission**  **(n=1602/744/858)** |  |  |  | <0.001 |
| Septic shock / sepsis | 225 (14.0) | 101 (13.6) | 124 (14.5) |  |
| Other shock | 106 (6.6) | 59 (7.9) | 47 (5.5) |  |
| Severe trauma | 57 (3.6) | 37 (5.0) | 20 (2.3) |  |
| De novo acute respiratory failure | 378 (23.6) | 103 (13.8) | 275 (32.0) |  |
| Acute or chronic respiratory failure | 168 (10.5) | 40 (5.4) | 128 (14.9) |  |
| Coma/Seizures | 166 (10.4) | 100 (13.4) | 66 (7.7) |  |
| Metabolic disorders | 77 (4.8) | 53 (7.1) | 24 (2.8) |  |
| Cardiac arrest | 60 (3.7) | 30 (4.0) | 30 (3.5) |  |
| Post-operative surveillance | 202 (12.6) | 106 (14.3) | 96 (11.2) |  |
| Other | 163 (10.2) | 115 (15.5) | 48 (5.6) |  |
| **Admission category**  **(n=1601/744/857)** |  |  |  | 0.005 |
| Medical | 1204 (75.2) | 529 (71.1) | 675 (78.8) |  |
| Scheduled surgery | 159 (9.9) | 83 (11.2) | 76 (8.9) |  |
| Urgent surgery | 206 (12.9) | 114 (15.3) | 92 (10.7) |  |
| Trauma | 32 (2.0) | 18 (2.4) | 14 (1.6) |  |
| **SAPS II (n=1557/725/832)** | 40 [29-55] | 37 [26-52] | 43 [31-57] | <0.001 |
|  |  |  |  |  |
| **Chronic respiratory disease (obstructive) (n=1599/744/855)** | 419 (26.2) | 131 (17.6) | 288 (33.7) | <0.001 |
| **Chronic respiratory disease (restrictive) (n=1599/744/855)** | 105 (6.6) | 34 (4.6) | 71 (8.3) | 0.003 |
| Obstructive sleep apnea syndrome  **(n=1599/744/855)** | 123 (7.7) | 52 (7.0) | 71 (8.3) | 0.33 |
| Chronic oxygen therapy **(n=1598/743/855)** | 91 (5.7) | 20 (2.7) | 71 (8.3) | <0.001 |
| **Long-term non-invasive ventilation**  **(n=1599/744/855)** | 44 (2.8) | 12 (1.6) | 32 (3.7) | 0.009 |
| **Chronic heart failure**  **(n=1599/744/855)** | 252 (15.8) | 107 (14.4) | 145 (17.0) | 0.16 |
| **Chronic kidney failure (n=1599/744/855)** | 166 (10.4) | 83 (11.2) | 83 (9.7) | 0.34 |
| **Cirrhosis (n=1599/744/855)** | 94 (5.9) | 49 (6.6) | 45 (5.3) | 0.26 |
| **Cancer (n=1599/744/855)** | 171 (10.7) | 92 (12.4) | 79 (9.2) | 0.04 |
| **Immunosuppression (n=1596/743/853)** | 187 (11.7) | 88 (11.8) | 99 (11.6) | 0.88 |

**Additional file 1: Table S3. ARDS exclusion criteria among patients with bilateral infiltrates**

|  | Total n=228^a^ |
| --- | --- |
| **Respiratory support not include in the ARDS definition** | 96 (42.1) |
| Ambiant Air | 3 (1.3) |
| Low flow oxygen | 65 (28.5) |
| High flow oxygen | 28 (12.3) |
| **PEEP <5cm H2O under NIV (n=28)^b^** | 2 (0.9) |
| **PEEP <5cm H2O under invasive ventilation (n=104)^c^** | 16 (7.0) |
| **Bilateral opacities fully explained by pleural effusions, lobar/lung collapse, or nodules** | 124 (54.4) |
| **Respiratory failure fully explained by cardiac failure or fluid overload** | 95 (41.7) |
| **> 1 week of new or worsening respiratory symptoms** | 75 (32.9) |

a total is higher than 100% as a single patient may have multiple exclusion criteria

b One missing value

c Three missing values

**Additional file 1: Table S4. ICU mortality according to ventilator support and hypoxemia class**

|  | **Ambient air**  **N=14** | **Low flow oxygen**  **N=190** | **High flow oxygen**  **N=45** | **NIV**  **N=83** | **IV**  **N= 511** |
| --- | --- | --- | --- | --- | --- |
| **ICU death /**  **Nb P/F 200-300 (%)** | **1/11 (9)** | **14/135 (10)** | **2/11 (18)** | **4/43 (9)** | **71/233 (30)** |
| **ICU death /**  **Nb P/F 100-200** | **2/3 (67)** | **8/55 (15)** | **2/23 (9)** | **8/32 (25)** | **76/224 (34)** |
| **ICU death /**  **Nb P/F ≤ 100** | **0/0 (-)** | **0/0 (-)** | **3/11 (27)** | **2/8 (25)** | **32/54 (59)** |

**Additional file 1: Table S5. Univariate analysis of variables associated with mortality in hypoxemic patients**

|  | HR * [IC95%] | p |
| --- | --- | --- |
| **Age** | 1.02 [1.02-1.03] | <0.001 |
| Male | 1 (ref) | 0.236 |
| Female | 1.19 [0.89-1.60] |  |
| Body mass index | 0.98 [0.96-1.00] | 0.049 |
| Obesity | 0.74 [0.54-1.01] | 0.061 |
| **Main diagnosis at ICU admission** |  | <0.001 |
| Septic shock / sepsis | 1.25 [0.84-1.86] |  |
| Other shock | 2.30 [1.44-3.68] |  |
| Traumatism | 0.13 [0.02-0.97] |  |
| De novo acute respiratory failure | 1 (ref) |  |
| Acute on chronic respiratory failure | 1.27 [0.81-1.99] |  |
| Coma/Seizures | 1.24 [0.74-2.10] |  |
| Metabolic disorders | 1.23 [0.45-3.38] |  |
| Cardiac arrest | 1.95 [1.05-3.61] |  |
| Post-operative surveillance | 0.59 [0.30-1.16] |  |
| Other | 0.57 [0.28-1.14] |  |
| **Admission category** |  | 0.665 |
| Medical | 1 (ref) |  |
| Scheduled surgery | 0.63 [0.29-1.33] |  |
| Urgent surgery | 0.92 [0.61-1.39] |  |
| trauma | - |  |
| **SAPS II** | 1.02 [1.01-1.03] | <0.001 |
| Chronic respiratory disease (obstructive) | 1.06 [0.80-1.41] | 0.694 |
| Chronic respiratory disease (restrictive) | 1.84 [1.23-2.74] | 0.003 |
| Obstructive sleep apnea syndrome | 0.86 [0.57-1.31] | 0.481 |
| Chronic oxygen or non-invasive ventilation | 0.88 [0.55-1.40] | 0.589 |
| Chronic heart failure | 2.07 [1.50-2.85] | <0.001 |
| **Chronic kidney failure** | 1.67 [1.11-2.53] | 0.015 |
| **Cirrhosis** | 1.69 [1.02-2.82] | 0.043 |
| **Cancer** | 1.84 [1.22-2.78] | 0.004 |
| **Immunosuppression** | 1.94 [1.36-2.77] | <0.001 |
|  |  |  |
| **P/F ratio by 10 mmHg decrease** | 1.05 [1.03-1.07] | <0.001 |
| **Hypoxemia severity class** |  | 0.002 |
| Mild | 1 (ref) |  |
| Moderate | 1.30 [0.97-1.76] |  |
| Severe | 2.06 [1.38-3.06] |  |
| **ARDS** | 1.64 [1.23-2.18] | <0.001 |
| **Oxygen administration** |  | 0.395 |
| Ambient air | 1.80 [0.54-6.00] |  |
| Low-flow oxygen | 1 (ref) |  |
| High-flow oxygen | 1.07 [0.46-2.48] |  |
| Non-invasive ventilation | 1.49 [0.69-3.20] |  |
| Invasive ventilation | 1.49 [0.97-2.30] |  |
| **Vasopressors** | 1.46 [1.01-2.11] | 0.044 |
| **Renal replacement therapy during ICU stay** | 1.55 [1.17-2.04] | 0.002 |
| **Prone positioning** | 1.19 [0.58-2.42] | 0.638 |
| **Inhaled NO** | 2.49 [1.31-4.73] | 0.005 |
|  |  |  |

***Univariate Cox model**

**Additional file 1: Table S6. aHRs of PaO2 / FiO2 banding in different sensitivity analyses**

|  | aHR * [IC95%] | p |
| --- | --- | --- |
| **Model 1 : including ventilator support in the model** |  |  |
| Oxygenation modalities |  | 0.630 |
| Ambient air | 1 (ref) |  |
| Low-flow oxygen | 0.98 [0.25-3.78] |  |
| High-flow oxygen | 0.85 [0.20-3.63] |  |
| Non-invasive ventilation | 1.75 [0.42-7.38] |  |
| Invasive ventilation | 1.11 [0.31-3.96] |  |
| Hypoxemia class |  | <0.001 |
| Mild | 1 (ref) |  |
| Moderate | 1.37 [0.98-1.90] |  |
| Severe | 2.64 [1.67-4.17] |  |
|  |  |  |
| **Model 2 : only in patients with actual PaO2 / FiO2** |  |  |
| Hypoxemia class |  | 0.001 |
| Mild | 1 (ref) |  |
| Moderate | 1.17 [0.80-1.71] |  |
| Severe | 2.43 [1.49-3.97] |  |
|  |  |  |
| **Model 3 : excluding patients on chronic oxygen therapy or NIV** |  |  |
| Hypoxemia class |  | <0.001 |
| Mild | 1 (ref) |  |
| Moderate | 1.34 [0.96-1.89] |  |
| Severe | 2.58 [1.58-4.21] |  |
|  |  |  |
| **Model 4 : excluding patients with therapeutics withholding/withdrawing** |  |  |
| Hypoxemia class |  | 0,008 |
| Mild | 1 (ref) |  |
| Moderate | 1.41 [0.96-2.08] |  |
| Severe | 2.46 [1.38-4.37] |  |
|  |  |  |

* adjusted Hazard ration obtained using 4 different multivariate Cox models

**Additional file 1: Figure S1.** **Evolution of oxygen support after the day of study**


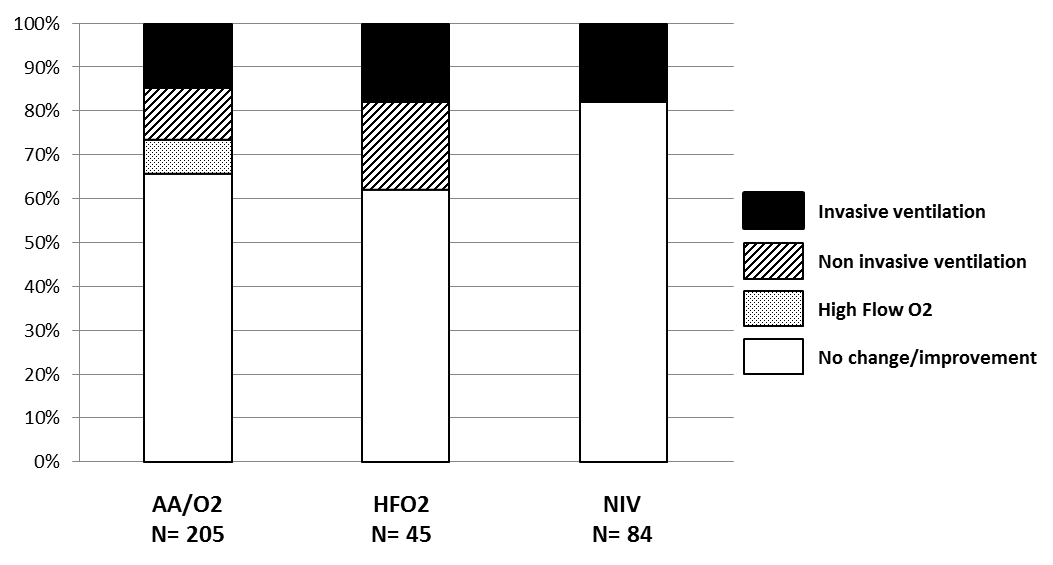


Maximal invasive oxygen support after the day of study. The graphic depict the rate of patients with a more invasive oxygen support after the day of study. Air ambient and low flow O2 have been pooled. AA/O2: Air ambient/low flow O2. HFO2 High Flow oxygenotherapy, NIV Non-invasive ventilation.
